# Native Mass Spectrometry: What is in the Name?

**DOI:** 10.1007/s13361-016-1545-3

**Published:** 2016-12-01

**Authors:** Aneika C. Leney, Albert J. R. Heck

**Affiliations:** 1 Biomolecular Mass Spectrometry and Proteomics, Bijvoet Center for Biomolecular Research and Utrecht Institute for Pharmaceutical Sciences, Utrecht University, Padualaan 8, 3584CH Utrecht, The Netherlands; 2 Netherlands Proteomics Center, Padualaan 8, 3584CH Utrecht, The Netherlands

**Keywords:** Electrospray ionization mass spectrometry (ESI-MS), Native mass spectrometry

## Abstract

Electrospray ionization mass spectrometry (ESI-MS) is nowadays one of the cornerstones of biomolecular mass spectrometry and proteomics. Advances in sample preparation and mass analyzers have enabled researchers to extract much more information from biological samples than just the molecular weight. In particular, relevant for structural biology, noncovalent protein–protein and protein–ligand complexes can now also be analyzed by MS. For these types of analyses, assemblies need to be retained in their native quaternary state in the gas phase. This initial small niche of biomolecular mass spectrometry, nowadays often referred to as “native MS,” has come to maturation over the last two decades, with dozens of laboratories using it to study mostly protein assemblies, but also DNA and RNA-protein assemblies, with the goal to define structure–function relationships. In this perspective, we describe the origins of and (re)define the term native MS, portraying in detail what we meant by “native MS,” when the term was coined and also describing what it does (according to us) not entail. Additionally, we describe a few examples highlighting what native MS is, showing its successes to date while illustrating the wide scope this technology has in solving complex biological questions.

**Graphical Abstract:**
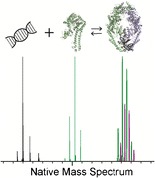
ᅟ

ᅟ

## Introduction

All cellular biological processes involve the complex coordination of proteins and other biomolecules (e.g., DNA, RNA, lipids, metabolites) in space and time. To facilitate this, proteins often form physical albeit dynamic complexes or networks, enabling them to work efficiently together to carry out their function. Increased fundamental knowledge on these complex cellular interactomes is crucial for not only mapping novel interactions [1] but also for drug design [2] and integrating novel technologies for disease diagnostics [3, 4]. Mass spectrometry (MS), specifically MS-based proteomics, has already played a pivotal role in deciphering the many protein complexes and protein interaction networks that are present in the cell, typically at the end of a pipe-line that starts with the immune-purification of a tagged-bait protein, followed by identification of the co-purified proteins using LC-MS/MS [5, 6]. Although rich in information in terms of the number of protein complexes this type of LC-MS/MS analysis can identify, obtaining structural details such as subunit stoichiometry, topology, and structural models in atomic detail by these means is challenging. It is to this end that traditional biophysical techniques such as X-ray crystallography, NMR, electron microscopy [7] and small angle scattering [8] excel. However, not all systems are amenable for analysis by these techniques.

Thus, different methods of mass spectrometry (compared with MS-based proteomics analysis) are rapidly emerging that are complimentary to the aforementioned biophysical techniques for structural biology, enabling investigations into tertiary and quaternary structures of protein assemblies. These span the range from hydrogen/deuterium exchange mass spectrometry, chemical surface labeling techniques, cross-linking mass spectrometry, up to the direct mass analysis of intact protein assemblies [9]. The latter arose following initial work by a few laboratories in the 1990s [10, 11], demonstrating that noncovalent interactions could be preserved in the gas phase for analysis, enabling information on subunit stoichiometry, binding partners, protein complex topology, protein dynamics, and even binding affinities from a single mass spectrometric analysis [12–17]. Cumulatively, these endeavours have been later coined by the term “native mass spectrometry,” which now has become a commonly accepted term for these activities as evidenced also by the name of the 31st ASMS Asilomar Conference, “Native Mass Spectrometry-Based Structural Biology” held in the autumn of 2015.

## What is Native MS?

Native MS is a particular approach based on electrospray ionization, whereby the biological analytes are sprayed from a nondenaturing solvent. The term native MS was coined in 2004 [15]; however, it described approaches that had been introduced earlier [10, 18–20]. In these pioneering studies, terms like nondenaturing, macromolecular, or supramolecular MS were used to describe native MS. Even today, terminologies such as noncovalent MS, native spray MS, or simply electrospray ionization-MS are frequently used to describe this specific type of biological MS analysis. The use of multiple names can be confusing to the broader scientific audience; therefore, by (re)defining herein native MS in detail, we encourage researchers to unify and further adopt the term native MS.

Some confusion around the definition of native MS has always existed, in and outside the mass spectrometry community, likely originating from the fact that “native” in its purest definition would mean the state of a protein in its natural environment (i.e., within the cell, in its natural location in the vicinity of its natural molecular partners). Native MS being a gas-phase method certainly cannot live up to that definition.

The term “native” in native MS describes the biological status of the analytes in solution, *prior* to the ionization event. During native MS analysis, therefore, it is imperative that researchers carefully control parameters such as pH and ionic strength to maintain the native folded state of the biological analytes in solution. Owing to these measures, information can be inferred by native MS that describes the nature of biological complexes in solution. Yet, during native MS the analyte of interest is per definition not in its native state at the point of detection, being inside the vacuum of the mass analyzer (i.e., after transition into the gas phase). Moreover, native MS, as we defined it above, even comprises ion mobility and/or tandem MS experiments whereby individual protein subunits are released in the gas phase from large macromolecular complexes to determine overall complex topology, since the protein complex was in its native state in solution *prior* to MS analysis.

One can compare the native MS terminology to that used in similar biophysical techniques such as polyacrylamide gel electrophoresis (PAGE) and immunoprecipitation [21]. In PAGE, proteins are separated based on their size and charge. Two types of PAGE conditions are typically used; denaturing PAGE whereby samples are typically reduced and heat denatured prior to analysis, and native PAGE whereby the analytes’ natural quaternary structure is maintained [22]. Likewise, the term native is also used to describe certain immunoprecipitation assays [23, 24]. Here, proteins and their interactors are typically pulled down with antibodies from cell lysates, the protein–antibody complexes are then disrupted, and the released proteins analyzed by techniques such as denaturing PAGE, LC-MS, or Western blotting. Thus, comparable with the use of tandem MS to determine complex topology with native MS, the protein complexes analyzed by native immunoprecipitation are not native at the point of detection but, more importantly, the biological analytes are in their native-like state in solution prior to further analysis, enabling the information obtained to reveal structural details about the protein complexes of interest.

Furthermore, native MS inherently is a technique for in vitro analysis, whereby we can only mimic as best the biological environment in which proteins and their complexes exist in vivo in their cellular environment. But native MS is not unique in that as in nearly all structural biology techniques, the biomolecules are removed from their cellular habitat, thus also with all these techniques the term native should be taken lightly.

Finally, it is important to note that although proteins and protein complexes are the most commonly studied analytes by native MS to date, and thus are the main focus of this perspective, native MS can also be used to describe the analysis of a variety of macromolecular assemblies, such as ribonucleoprotein complexes (e.g., the ribosome [25]), protein–lipid complexes such as transport channels [26], nucleic acid structures such as DNA G-quadruplexes [27], and noncovalent drug–nucleic acids interactions [28]. Moreover, we foresee that this area of MS analysis will only continue to expand as, for instance, the analysis of RNA and DNA tertiary structures is still complex and much less explored [29].

## Key Milestones in Native MS

**Figure 1:**
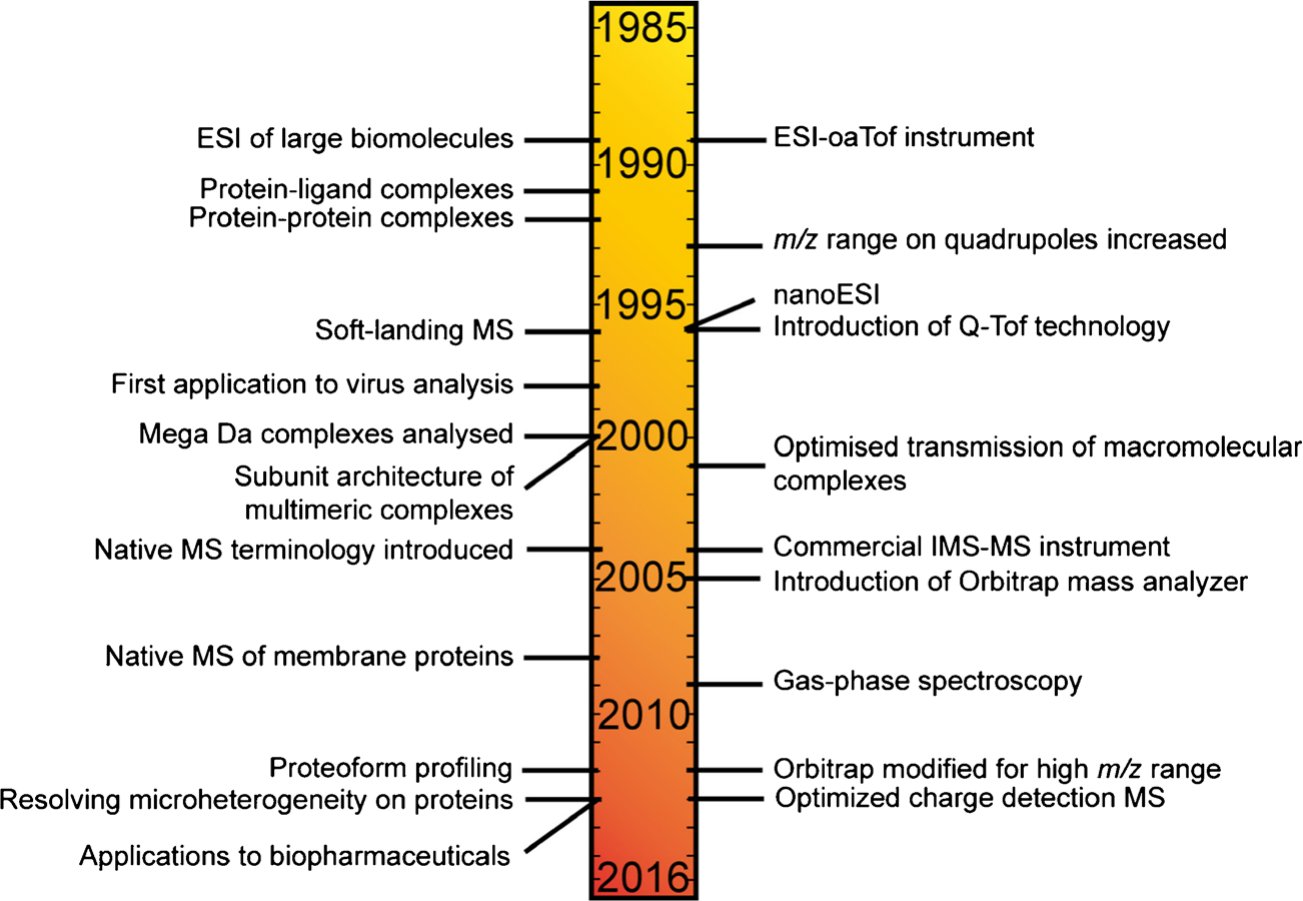
Timeline and origin of native MS. Key instrumental developments (right) and biological applications (left) of native MS over the last three decades

Up to the 1980s, MS was almost solely used to measure the mass to charge ratio (*m*/*z*) of small organic molecules (Figure 1). Mass analyzers provided sufficiently high resolution and mass accuracy of small molecules that when combined with tandem MS could determine the elemental composition of the compound of interest. It was not until the revolutionary development of soft ionization methods, such as electrospray ionization (ESI) [30] and matrix assisted laser desorption ionization (MALDI) [31], that the power of MS in terms of the information it could provide on larger biomolecules was realized. In MALDI, the analyte of interest is co-crystalized with IR- or UV-absorbing organic acids and laser ablation used to transfer the sample into the gas phase for analysis. Although successful for single protein analysis, preserving large macromolecular complexes in the (often acidic and denaturing) matrix used for MALDI can be problematic. In addition, the singly charged ions formed can have high *m*/*z* values that span beyond the range of most mass analyzers, notwithstanding notable exceptions [32, 33]. In the nowadays more popular ionization technique for biomolecules, ESI [34], analytes in solution are passed through a capillary whereby a high voltage is applied. A mist of highly charged droplets is formed; these droplets are reduced in size through coulombic fission events as they travel down a potential and pressure gradient through the inlet of the mass spectrometer towards the high-vacuum of the analyzer. Owing to the ionization mechanism, ESI produces multiply charged protein ions that circumvents the problems associated with detecting singly charged ions, whilst simultaneously enabling proteins to be analyzed directly from solution. Since its introduction to large molecule analysis in 1989 by Fenn et al. [30], ESI has become increasingly specialized for biomolecule analysis. Coated glass capillaries are now used whereby the orifice size is 1–5 μm in diameter, enabling droplet size to be reduced by an order of magnitude compared with traditional ESI. This lower flow rate ESI, termed nanoESI [35], is highly advantageous for native MS analysis because of its low sample consumption, more uniform response factors, and higher tolerance to salts and buffers. Thus, nanoESI is now by far the most commonly used ionization method in native MS.

An equally important advancement in native MS analysis came with the developments in mass analyzers. Early studies used triple quadrupole mass analyzers, which had a maximum acquisition range of typically 2000, sometimes 4000 *m*/*z*. This *m*/*z* range was subsequently increased by the introduction of quadrupoles that operated at a lower radiofrequency [36, 37]; however, the high *m*/*z* range was compromised by a lower resolving power. On the other hand, time-of-flight (ToF) mass analyzers have no theoretical upper mass range, and can achieve high sensitivity and resolution on a fast timescale [38]. Thus, next work in native MS focused on the combination of nanoESI with ToF analyzers. One key pioneering example was the analysis of the enzyme 4-oxalocrotonate tautomerase [39], which upon ESI-MS analysis confirmed the oligomeric status of the enzyme, one of the leading examples at the time that native protein structures could be preserved into the gas phase for analysis. With the knowledge that noncovalent protein complexes could be analyzed, Sciex and Micromass Ltd. modified their quadrupole (Q)-ToF mass spectrometers [40, 41], which together with elegant work on the optimization and transmission of high *m*/*z* ions [42, 43], enabled tandem MS experiments to be performed on large protein complexes, providing information not only on protein complex stoichiometry but also on protein complex topology [41, 44, 45]. The Q-ToF technology rapidly became the platform most successful for native MS analysis, whereby the samples amendable for mass analysis reached sizes/molecular weights of over several million Daltons [46]. Despite these significant instrumental advancements, native MS in the early 2000s was still limited by the solubility of proteins/protein complexes in aqueous solution. Thus, for instance, native MS analysis of membrane proteins that require detergents for solubilization in aqueous buffers was still highly challenging. A significant breakthrough in membrane protein analysis, however, came with the realization that membrane proteins could be released intact from detergent micelles in the gas phase [47]. In the work by the Robinson group, *n*-dodecyl-β-D-maltoside micelles were used to protect the heteromeric adenosine 5′-triphosphate-binding cassette transporter BtuC_2_D_2_ upon transition from solution into the gas phase, whereby the intact mass of both the intact complex and dissociated subunits could be measured [47]. Nowadays, with the application of alternative solubilization techniques such as Amphipols [48] and Nanodiscs [49], the analysis of membrane proteins by native MS has become more widespread with studies of over 30 membrane proteins/membrane protein complexes being reported to date.

With the growing demand for MS analysis of macromolecular complexes both in the area of structural biology and in the biopharmaceutical industry [50], the need for more sensitive, higher resolution instruments increased. Addressing these issues, an Orbitrap mass analyzer [51] modified for the transmission of ions in the high *m*/*z* range was introduced for native MS analysis in 2012 [52]. With increased sensitivity, analyzer resolution, and enhanced desolvation, the achievable resolving power on proteins and protein assemblies using the Orbitrap mass analyzer increased substantially in comparison with traditional Q-ToF instruments, especially in the higher *m*/*z* range (*m*/*z* > 4000). This increased resolving power has enabled small differences in proteoforms, such as glycosylation on antibodies [53, 54] and phosphorylation on proteins and protein complexes [55, 56], to be more readily resolved. One striking example is the analysis of erythropoietin, whereby 236 glycan proteoforms were separated by *m*/*z* and identified [57]. As such, the Orbitrap mass analyzer is rapidly becoming on par with the Q-ToF for native protein MS analysis, with both instruments having their own strong niches today.

Of final note, charge detection MS (CDMS) has also expanded the range of protein complexes that can be analyzed by MS [58]. Being able to monitor both charge and *m*/*z* of single ions, CDMS provides significant advances over Q-ToF and Orbitrap mass analyzers alone, whereby at the current stages of development the charge state of complexes over several hundred kDa in size are too heterogeneous to decipher [59].

## Protein Structure in the Gas Phase

**Figure 2:**
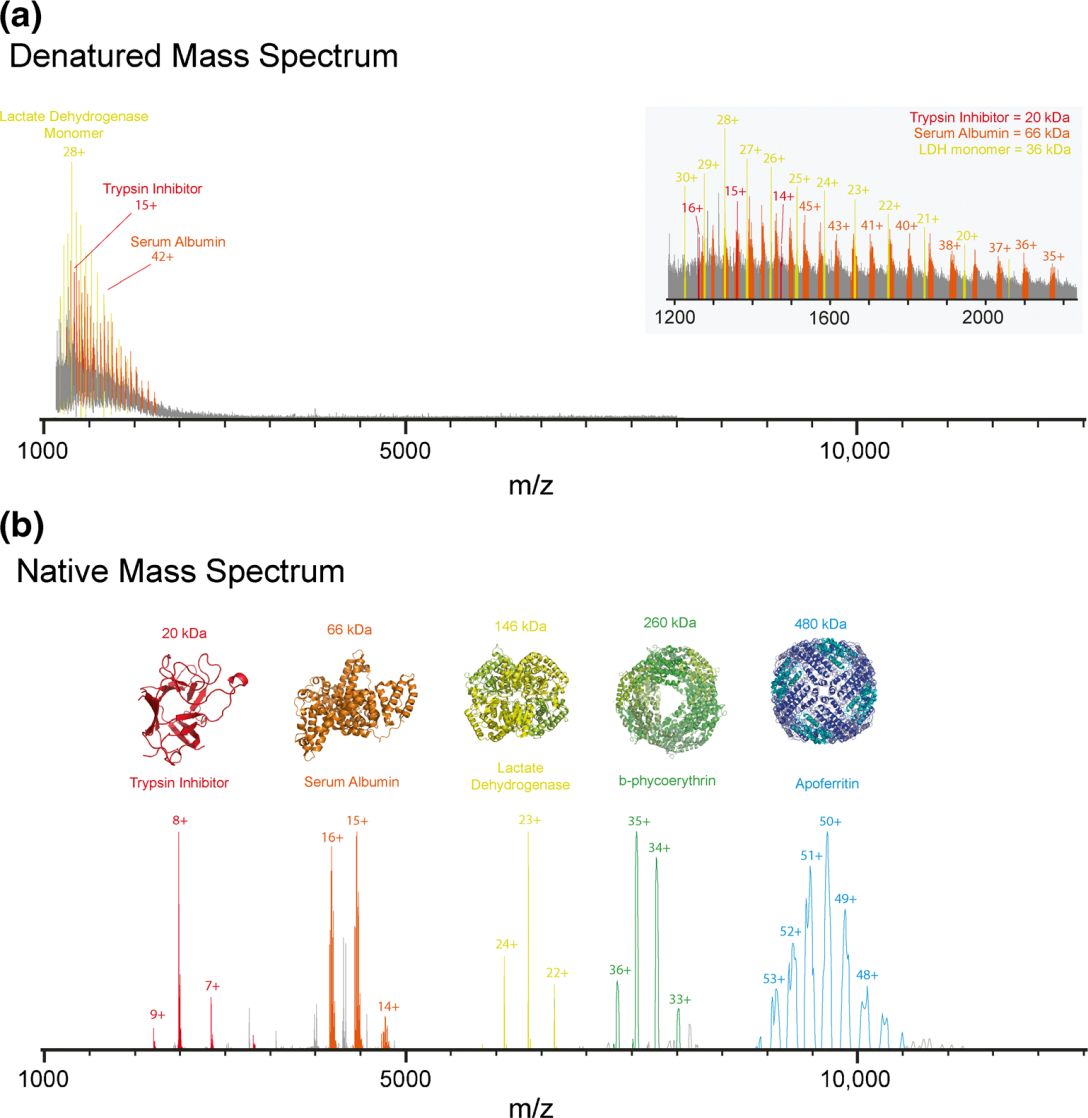
Comparison of the MS analysis of an identical protein mixture under denatured and native MS conditions. A mixture of proteins, trypsin inhibitor, bovine serum albumin, lactate dehydrogenase, B-phycoerythrin, and apoferritin, commonly used as molecular weight markers in native PAGE, was buffer-exchanged into a water/acetonitrile/formic acid (50/49/1) solution or 100 mM aqueous ammonium acetate pH 7 for denatured MS (**a**) and native MS (**b**), respectively. For native MS analysis, the MS parameters on the Orbitrap EMR were optimised specifically for the *m*/*z* window of each protein/protein complex and the mass spectra “stitched” together. The proteins corresponding to trypsin inhibitor, bovine serum albumin, lactate dehydrogenase, B-phycoerythrin, and apoferritin are highlighted in red, orange, yellow, green, and blue, respectively. Native MS shows all proteins and protein complexes are widely separated in *m*/*z* space, in sharp contrast to denaturing MS where the *m*/*z* of the ions corresponding to all the proteins and protein subunits are collapsed in the narrow 1000–2500 *m*/*z* range

As previously stated, the term “native” in native MS refers only to the biological status of the protein/biological assembly in solution *prior* to mass analysis. Notably, there is still, also within the MS community, a heated debate over to what extent native protein structures can be preserved in the gas phase. Initial evidence that protein structure could be partly maintained in the gas phase came from the observation that proteins electrosprayed from aqueous solution exhibited narrower charge state distributions (charge envelopes) compared with proteins sprayed from organic solvents. This observation was rationalized by the fact that during the ESI process, assuming the charge residue model [60], only solvent exposed basic residues could pick up a charge/proton. Thus, an unfolded/less structured protein in its most expanded form (i.e., in acidified organic solvent) will pick up more charges during ESI than a folded/more structured protein (i.e., electrosprayed from aqueous solution). This is exemplified in Figure 2 whereby the most abundant charge state for bovine serum albumin is 15+ in aqueous buffer in contrast to 50+ when the bovine serum albumin was sprayed denatured from a 1:1 mixture of water:acetonitrile containing 1% formic acid.

Although the observation that charge state distributions reflect protein conformation was revolutionary at that time, more evidence was needed to convince structural biologists that the tertiary structure of proteins could be partly maintained in the gas phase. One early study by Siuzdak et al. addressed this issue by using soft landing experiments whereby the tobacco mosaic virus was electrosprayed from solution and subsequently captured and visualized by transmission electron microscopy [61]. Interestingly, this virus was structurally still viable, proven by its ability to infect tobacco plants after its flight through the mass spectrometer, highlighting that the virus capsid structure survived upon the transition into the gas phase. Perhaps even more convincing arguments that protein structures can be partly retained in the gas phase come from more recent ion mobility spectrometry (IMS) experiments [62, 63]. IMS separates ions based on their shape and charge. Thus, combined with native MS, IMS can provide information on collision cross-sections of proteins in the gas phase that can be directly compared with in-solution measurements of cross-sections obtained by other biochemical techniques such as X-ray crystallography, dynamic light scattering, or electron microscopy. Some of the first IMS analysis of small proteins such as ubiquitin, cytochrome *c*, and myoglobin showed that the cross-sections measured in the gas-phase electrospray from aqueous solvent were smaller than those electrosprayed from organic solvent and were often consistent with the values predicted based on crystallographic data [64–66]. Since then, IMS-MS has been successfully applied to monitor protein conformational changes [67, 68] and to gain information on how proteins assemble/misassemble into large macromolecular complexes [69–71].

The majority of the proteins studied by native MS to date have been shown to at least partly retain native-like structures in the gas phase. Exceptions do exist, whereby proteins either unfold in the gas phase because of coulombic repulsion [72] or, alternatively, form more compact partly collapsed structures, a phenomenon termed gas-phase collapse [73]. For example, cross-sectional measurements of GroEL, a chaperonin consisting of two heptameric rings, were reported to be substantially smaller than that measured by crystallography data [73]; the difference was attributed to the collapse of the ring structures in the gas phase. Coulombic repulsion, on the other hand, has been reported for the protein GB1 using gas-phase Förster resonant energy transfer (FRET), a sensitive method that acts as a “spectroscopic ruler” measuring the distance between defined amino acids within a protein sequence [72]. Here, the FRET efficiency decreased with increasing charge state, indicative of protein unfolding. Interestingly, a lower FRET efficiency for all charge states of GB1 was reported in the gas phase, compared with in-solution measurements showing that the protein conformation in the gas phase is differential to its solution state [72]. Thus, much care still needs to be taken to avoid over-interpretation of native MS data since protein conformation can change upon transition from solution into the gas phase. On the other hand, it has been argued that some proteins could be more native-like in the gas phase compared with in aqueous solution. For example, the gas phase may be a highly suitable environment to study proteins that reside in very hydrophobic environments such as the membranes of cells.

Overall, although the debate whether native protein structure is maintained in the gas phase continues, the future is bright for structural MS analysis of proteins and their complexes, as illustrated by a growing body of work of, for instance, protein–lipid [26, 74, 75], protein–RNA [76], protein–DNA [77], protein–drug [78], and protein–protein complexes [79, 80].

## Native MS as a Complimentary Technique for Structural Biologists

A relatively simple illustrative example of native MS and its application potential in structural biology is depicted in Figure 2. Here, a mixture of proteins/proteins complexes that are commonly used as a molecular weight marker in native PAGE were analyzed by both denaturing and native MS. The protein mixture contained two proteins, trypsin inhibitor and bovine serum albumin, and three protein complexes, namely lactate dehydrogenase, B-phycoerythrin, and apoferritin, the molecular weights of which range from 20 to 480 kDa. Upon ESI-MS analysis of the protein mixture under denaturing conditions (50:49:1 water:acetonitrile:formic acid), a broad nearly unresolvable range of peaks at low *m*/*z* range is observed (Figure 2a). Detailed analysis revealed multiple peaks that could be attributed to trypsin inhibitor, serum albumin, and the lactate dehydrogenase monomer. However, although present in solution, peaks corresponding to the individual subunits of B-phycoerythrin and apoferritin were harder to assign. In sharp contrast, native MS analysis of the same mixture shows that all the five proteins/protein complexes are nicely separated in *m*/*z* space (Figure 2b). In addition, since the only ions that were detected corresponded to the intact molecular weights of the protein complexes, all the noncovalent complexes in solution were preserved on transition into the gas phase for analysis revealing information on the protein complex mass and, thus, stoichiometry. Multiple species were observed for bovine serum albumin and apoferritin, showing the power of native MS in determining the micro-heterogeneity that may be present within proteins and their complexes.

**Figure 3:**
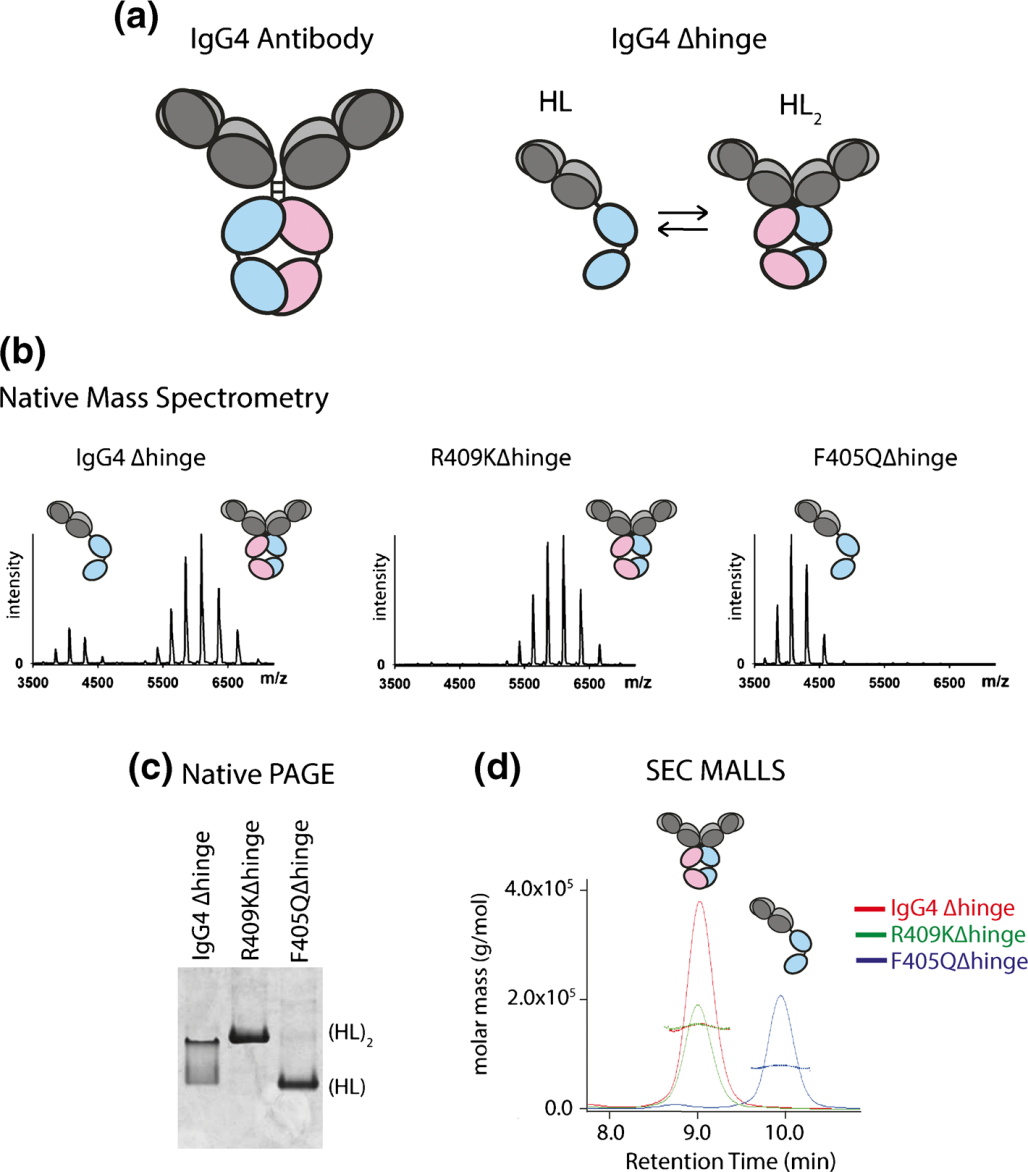
Native MS compares well with alternative methods to probe quaternary structures. (**a**) Cartoon of the wild-type IgG4 antibody and an IgG4 antibody whereby the hinge region is deleted (IgG4Δhinge). IgG4Δhinge exists in solution in equilibrium between its monomeric (HL) and dimeric (HL_2_) state. (**b**) Native MS of three HL mutants; IgG4Δhinge, R409KΔhinge, and F405QΔhinge. Two charge state distributions are observed for IgG4Δhinge corresponding to its monomeric and dimeric state, well separated in *m*/*z* space. In contrast, predominantly the monomeric and dimeric states were observed for the IgG4Δhinge variants F405Q and R409K, respectively. (**c**) Native PAGE of IgG4Δhinge, R409KΔhinge, and F405QΔhinge, showing different band migrations corresponding to the oligomeric status (i.e., monomeric/dimeric) of the variants in solution. (**d**) Size exclusion chromatography (SEC) multi-angle laser light scattering (MALLS) of IgG4Δhinge (red), R409KΔhinge (green), and F405QΔhinge (blue), showing the difference in retention times/molar masses measured reflecting their either monomeric, dimeric, or mixed status in solution [81]. The figure is adapted from [81]

Native MS can be used to interrogate the heterogeneity present within protein samples, thus placing it in a privileged position over NMR spectroscopy and X-ray crystallography that mostly report on pure proteins/protein complexes. Alternatively, other biophysical techniques, such as size exclusion chromatography combined with multi-angle laser light scattering (SEC MALLS) and native PAGE can provide complementary information to native MS, all being capable of separating protein mixtures according to their size, albeit at lower resolving power than native MS. One example of the complementarity of these techniques was highlighted during an investigation into the effect of single mutations on antibody half molecules (HL), whereby the hinge region of IgG4 was deleted (IgG4Δhinge) [81] (Figure 3a). After removal of the hinge region, HL are present in an equilibrium between their monomeric and dimeric states [82]. Mutations in the constant domain (CH3) on the heavy chain can substantially alter this equilibrium, increasing (for example, in the case of R409K) or decreasing (for example, in the case F405Q) the binding affinity between the CH3 domains in the dimeric HL_2_ complex [81]. Native MS proved to be an ideal technique for evaluating this binding equilibrium qualitatively and even quantitatively. Figure 3b shows the native MS data acquired for the IgG4Δhinge WT, R409KΔhinge, and F405QΔhinge variants. The ratio of monomer:dimer observed differed between the variants, consistent with their differential K_D_ values measured; R409KΔhinge was predominantly dimeric, F405QΔhinge monomeric, and both oligomeric states were observed for IgG4Δhinge WT. Native PAGE (Figure 3c) and SEC MALLS (Figure 3d) on the same IgG4Δhinge variants was also performed whereby the band position and retention time/molar mass measured for each technique, respectively, differed for each variant, again consistent with its oligomeric status in solution. Thus, native MS provides complementarity information to other biochemical techniques, making it highly suitable for the structural characterization of proteins and their complexes, and the qualitative and even sometimes quantitative analysis of binding affinities [14, 83]. For the latter, careful analysis is required as nonspecific association may occur during the ionization process for some systems, especially when the analyte concentrations are high and/or the binding affinities low [84, 85].

## Future of Native MS

The majority of native MS analyses to date have been performed on recombinant proteins, for instance purified from expression systems, such as *Escherichia coli* or *CHO* cells. Such overexpressed recombinant proteins are typically of high purity, making them readily amenable to MS, X-ray, or NMR analysis. However, being removed from their natural environment, e.g., the human cell, they also are not analyzed in their true native state. For example, recombinant proteins overexpressed in bacteria are rarely post-translationally modified, a process that can be imperative for protein function, especially in eukaryotic systems. In addition, many proteins require binding partners, without the knowledge of which the proteins could have been analyzed in their improper oligomeric state or binding status. Thus, in native MS, which is relatively sensitive, for instance compared with NMR, efforts are now turning to focus on the analysis of endogenous protein complexes. Tandem affinity purification [86] techniques are continuing to improve, and thus the challenges associated with extracting pristine cell-derived assemblies in high enough concentrations for native MS analysis are slowly being overcome. One recent fine example is the analysis of the Nup84 subcomplex (the outer rings of the yeast nuclear pore complex, NPC) whereby a hetero–hexameric complex comprising subunits Nup84, Nup85, Nup145C, Nup120, Seh1, and Sec13 was successfully analyzed by native MS and its stoichiometry determined.

The wish and/or the aim for the future in native MS is now to make the analysis as easy, sensitive, and efficient as of current affinity-purification MS experiments, wherein in a matter of weeks several thousands of different bait-proteins within the cell could be tagged, affinity purified with their interactors, and analyzed by MS-based proteomics technologies, providing data on over 28,000 protein–protein interactions on more than 400 protein complexes [5, 6]. We are still far from that but, if successful, it will provide the next level of important structural information, such as stoichiometry, topology, and first structural models of the complexes under study.

The future of structural biology is hybrid [87, 88], indicating that the ideal studies on protein structure combine data of electron microscopy, X-ray crystallography, NMR spectroscopy, and other methods. MS slowly but certainly becomes one of the pillars in such hybrid or integrative structural biology approaches. The future of MS-based structural biology is certainly also hybrid, as native MS should be and is being more and more combined with complementary methods such as top-down and middle-down proteomics [89], cross-linking MS, covalent labeling MS, and hydrogen/deuterium exchange MS [9].

In conclusion, here we (re)defined the term native MS as the mass spectrometric analysis of biomolecules that are prior to their ionization in their most native-like state. The usability, accessibility, and structural information obtained from native MS approaches is still expanding, providing detailed structural data of biomolecules in terms of their conformation, micro-heterogeneity, interaction partners, binding stoichiometry, subunit dynamics, (dis)assembly, and complex topology. Native MS still has a prosperous future.
